# Fruit Intake and Abdominal Aortic Calcification in Elderly Women: A Prospective Cohort Study

**DOI:** 10.3390/nu8030159

**Published:** 2016-03-10

**Authors:** Nicola P. Bondonno, Joshua R. Lewis, Richard L. Prince, Wai H. Lim, Germaine Wong, John T. Schousboe, Richard J. Woodman, Douglas P. Kiel, Catherine P. Bondonno, Natalie C. Ward, Kevin D. Croft, Jonathan M. Hodgson

**Affiliations:** 1 Royal Perth Hospital, School of Medicine and Pharmacology, University of Western Australia, Perth 6000, Western Australia, Australia; catherine.bondonno@uwa.edu.au (C.P.B.); natalie.ward@uwa.edu.au (N.C.W.); kevin.croft@uwa.edu.au (K.D.C.); jonathan.hodgson@uwa.edu.au (J.M.H.); 2 Centre for Kidney Research, Children’s Hospital at Westmead, Sydney 2145, New South Wales, Australia; joshua.lewis@sydney.edu.au (J.R.L.); germaine.wong@health.nsw.gov.au (G.W.); 3 School of Public Health, Sydney Medical School, University of Sydney, Sydney 2006, New South Wales, Australia; 4 Sir Charles Gairdner Hospital Unit, School of Medicine and Pharmacology, University of Western Australia, Perth 6009, Western Australia, Australia; richard.prince@uwa.edu.au (R.L.P.); wai.lim@health.wa.gov.au (W.H.L.); 5 Department of Endocrinology and Diabetes, Sir Charles Gairdner Hospital, Perth 6009, Western Australia, Australia; 6 Department of Renal Medicine, Sir Charles Gairdner Hospital, Perth 6009, Western Australia, Australia; 7 Park Nicollet Osteoporosis Centre and HealthPartners Institute, HealthPartners, Minneapolis, MN 55416, USA; scho0600@umn.edu; 8 Division of Health Policy and Management, University of Minnesota, Minneapolis, MN 55455, USA; 9 Centre for Epidemiology and Biostatistics, School of Public Health, Flinders University of South Australia, Adelaide 5042, South Australia, Australia; richard.woodman@flinders.edu.au; 10 Institute for Aging Research, Hebrew Senior Life, Beth Israel Deaconess Medical Centre, Harvard Medical School, Boston, MA 02215, USA; kiel@hsl.harvard.edu; 11 School of Biomedical Sciences and Curtin Health Innovation Research Institute, Curtin University Western Australia, Perth 6102, Australia; natalie.ward@curtin.edu.au

**Keywords:** apples, fruit, abdominal aortic calcification, atherosclerosis, cardiovascular disease

## Abstract

Cardiovascular disease (CVD) is the leading cause of death worldwide. There is a consistent inverse relationship between fruit intake with CVD events and mortality in cross-sectional and prospective observational studies, but the relationship of fruit intake with measurements of atherosclerosis in humans is less clear. Nutritional effects on abdominal aortic calcification (AAC), a marker for subclinical intimal and medial atherosclerotic vascular disease, have not been studied previously. The aim of this study was to examine the cross-sectional relationship of total and individual fruit (apple, pear, orange and other citrus, and banana) intake with AAC, scored between 0 and 24. The current study assessed baseline data for a cohort of 1052 women over 70 years of age who completed both a food frequency questionnaire assessing fruit intake, and underwent AAC measurement using dual energy X-ray absorptiometry. AAC scores were significantly negatively correlated with total fruit and apple intakes (*p* < 0.05), but not with pear, orange or banana intakes (*p* > 0.25). In multivariable-adjusted logistic regression, each standard deviation (SD; 50 g/day) increase in apple intake was associated with a 24% lower odds of having severe AAC (AAC score >5) (odd ratio OR): 0.76 (0.62, 0.93), *p* = 0.009). Total and other individual fruit intake were not associated with increased odds of having severe AAC. Apple but not total or other fruit intake is independently negatively associated with AAC in older women.

## 1. Introduction

Cardiovascular disease (CVD) is the leading cause of death worldwide. One of the most consistent relationships observed in cross-sectional and prospective observational lifestyle studies is that a diet rich in fruit is associated with a lower risk of CVD [1,2,3,4]. In a meta-analysis of 16 prospective cohort studies, the mean reduction in risk of CVD mortality was 5% for each additional serving of fruit per day [5]. A poor dietary pattern, including a low intake of fruit, is estimated to contribute to over 4% of the global burden of disease, around five million deaths worldwide. It is the third highest contributor to mortality after high blood pressure and smoking [6].

Several studies have explored the relationships of intakes of specific fruit, primarily apples and oranges, with CVD-related outcomes. Higher apple [7,8,9,10,11] and orange [10,12] intakes have been associated with lower risk of coronary heart disease and stroke. This may be due in part to their content of soluble fibre, vitamins (vitamin C), minerals (potassium and magnesium) and/or phytochemicals (flavonoids) [13]. In particular, apples and oranges are often major contributors to the intake of fibre [14] and specific flavonoids have been linked to benefits on CVD risk [15,16,17,18,19]. However, the type of fibre and the amount and structure of the flavonoids differs between these fruits [19,20], which could result in differential relationships with CVD risk.

While the association of fruit intake with CVD events and mortality is clear [2,21,22], the relationship of fruit intake with markers and measures of atherosclerosis in humans is less clear. Atherosclerosis, a thickening of the artery wall as a result of the accumulation of calcium and fat, is recognized as a fundamental contributor to CVD events and is major underlying contributor to CVD events in post-menopausal women. Abdominal aortic calcification (AAC), an emerging subclinical measure of atherosclerosis [23], corresponds to the degree of coronary calcification [24] and atherosclerosis in other arteries [25], and predicts CVD mortality [26,27]. The aim of this study was to examine the cross-sectional associations of total and individual fruit (apple, pear, orange and other citrus, and banana) intake with AAC in a cohort of women over 70 years of age.

## 2. Subjects and Methods

### 2.1. Participants

Subjects included in this study were originally recruited in 1998 to a 5-year, double-blind, randomized control trial of daily calcium supplementation to prevent osteoporotic fracture, the Calcium Intake Fracture Outcome Study (CAIFOS), which has been previously described [28]. Participants were recruited from the Western Australian general population of women aged over 70 years by mail, using the electoral roll which is a requirement of citizenship. Of the 5586 women approached, 1510 women were willing and eligible to participate in the study and of these 1500 women were recruited into the study. All participants were ambulant with an expected survival beyond 5 years and were not receiving any medication (including hormone replacement therapy) known to affect bone metabolism. Baseline disease burden and medications were comparable between these participants and the general population of similar age although the participants were more likely to be from higher socio-economic groups [29]. In the subsequent 5 years following inclusion in the study, participants received 1.2 g of elemental calcium as calcium carbonate daily or a matching placebo. The current study included participants from the CAIFOS study who completed a food frequency questionnaire at baseline (1998), which assessed apple and other fruit intake (*n* = 1456). In 1052 of these women, abdominal aortic calcification (AAC) was also measured at baseline (1998 or 1999). Informed consent was obtained and the study was approved by the Human Ethics Committee of the University of Western Australia.

### 2.2. Dietary Assessment

Baseline (1998) dietary intake was assessed using a validated semi-quantitative food frequency questionnaire (FFQ) developed by the Anti‑Cancer Council of Victoria [30]. The intake of energy and nutrients were estimated based on frequency of consumption and usual portion size [31]. Intakes of individual fruits were assessed using specific items on the FFQ and quantified in grams per day (g/day).

### 2.3. Assessment of Abdominal Aortic Calcification (AAC)

Measurements of AAC were collected during 1998 (baseline) and 1999 (Year 1). Following bone density measurement, an additional lateral spine image was captured to measure AAC. All AAC scores from 0 to 24 were derived from digitally enhanced lateral single-energy images of the thoraco-lumbar spine using a Hologic 4500A bone densitometry machine (Hologic, Bedford, MA, USA). A single experienced investigator read all images using the validated semi quantitative scoring system [32]. The AAC 24 scoring system scores aortic calcification relative to each vertebral height (L1–L4) and is scored as; 0 (no calcification), 1 (≤1/3 of the aortic wall), 2 (>1/3 to ≤2/3 of the aortic wall) or 3 (>2/3 of the aortic wall) for both the anterior and posterior aortic walls giving a maximum possible score of up to 24. Severity of AAC was then categorized using previously published groupings [33]: low (AAC score 0 or 1); moderate (AAC score 2–5); and severe (AAC score >5) by an experienced assessor.

### 2.4. Covariates

Baseline questionnaires were used to determine values for potential confounding variables including age, BMI, energy intake, energy expended in physical activity, alcohol consumption, previous type 2 diabetes, use of antihypertensive medication, use of cholesterol-lowering medication (statins), and current or previous smoking. Previous atherosclerotic vascular disease was determined from primary discharge diagnoses from hospital records (1980–1998) as described previously [34].

The participants provided their previous medical history and current medications verified by their General Practitioner. These data were coded using the International Classification of Primary Care-Plus (ICPC-Plus) method. This coding methodology allows aggregation of different terms for similar pathologic entities as defined by the International Classification of Disease coding system. These data were then used to determine the presence of pre-existing diabetes (T89001-90009). Smoking status was coded as non-smoker or ex-smoker/current smoker (there were only 3 current smokers) if a participant had ever consumed more than 1 cigarette per day for more than 3 months during the past.

Weight was assessed using digital scales with participants wearing light clothes and no shoes. Height was assessed using a stadiometer and the body mass index was calculated in kg/m^2^ at baseline.

For physical activity, the women filled in a validated questionnaire that allowed estimation of energy used during exercise in kJ/day with the use of published energy costs of specific activities [35,36]. The women were asked whether they participated in any sports, recreation, or regular physical activity. Women who answered “no” to this question scored zero, and women who answered “yes” were asked to list up to 4 sports, recreational activities, or forms of regular physical activity, including walking, which were undertaken in the past 3 months. Energy expenditure (in kJ/day) for these activities was calculated with the use of published energy costs. This measure of energy expenditure is associated with bone density [37]. Total energy and alcohol intake, nutrient intakes and flavonoid intakes were estimated from the FFQ.

### 2.5. Statistical Analysis

Analyses were undertaken using IBM SPSS Statistics version 21 (2012, IBM Corp, Armonk, NY, USA). Statistical significance was set at *p* < 0.05 for all tests. Descriptive data are presented as mean ± SD for continuous variables, as number (*n*) and percentage (%) for categorical variables and as median (x̃) (interquartile range (IQR)) for variables not normally distributed. As AAC and total fruit intake were not normally distributed, we used a non-parametric correlation test (Spearman’s correlation) to assess univariate associations between AAC, fruit intake (g/day) and demographic variables; the results are presented as (Spearman’s rho (*ρ*), *p*-value). To assess the differences in median values of AAC, and total fruit intake between groups based on binary clinical measures (e.g., atherosclerosis) we used a non-parametric *t*-test (Mann Whitney U test). We compared AAC scores across total fruit intake tertiles and apple intake tertiles using univariate ANCOVA, with Bonferroni adjustment for multiple comparisons. AAC was then split into two groups: not severe (0–5) and severe (>5) and logistic regression analysis was used to assess the OR (odds ratio) and 95% confidence interval for severe AAC for each standard deviation (SD) increase in fruit intake. Two models of adjustment were used for the univariate ANCOVA and logistic regression analyses: age-adjusted and multivariable-adjusted (age, BMI, energy intake, energy expended in physical activity, alcohol consumption, prevalent atherosclerotic vascular disease, previous type 2 diabetes, use of antihypertensive medication, use of cholesterol-lowering medication (statins), and current or previous smoking). We also assessed associations between AAC and fruit intake with additional adjustment for total vegetable intake and saturated fat intake, as a diet rich in fruit is associated with higher vegetable consumption [38] and lower consumption of saturated fat-rich food [39]. In addition, the potential mediating effects of dietary factors found in fruit that are linked to chronic disease protection was investigated using further adjustment for intakes of flavonoids, total dietary fibre, potassium, magnesium, and vitamin C. The OR and 95% confidence interval of severe abdominal aortic calcification for each SD increase in apple intake (50 g/day) was then stratified by BMI, health status and the use of medications. To assess if relationships of apple intake with AAC differed according to these parameters an interaction term was also included in the models.

## 3. Results

The baseline characteristics of the participants are shown in Table 1. At baseline, AAC score was positively correlated with age (*ρ* = 0.066, *p* = 0.032) and alcohol intake (*ρ* = 0.078, *p* = 0.011) and was significantly higher in women with prevalent atherosclerotic cardiovascular disease (2 (0–4) *vs.* 3 (1–6), *p* < 0.001)), taking antihypertensive medication (2 (0–4) *vs.* 2 (0–5), *p* = 0.011)), taking cholesterol-lowering medication (2 (0–4) *vs.* 3 (1–6), *p* < 0.001)) and in previous or current smokers (2 (0–4) *vs.* 3 (1–5), *p* < 0.001)). At baseline, total fruit intake (g/day) was positively correlated with energy intake (*ρ* = 0.23, *p* < 0.001) and physical activity (*ρ* = 0.087, *p* < 0.001), and negatively correlated with alcohol intake (*ρ* = −0.098, *p* < 0.001).

The univariate analysis of AAC score with total fruit and individual fruits are presented in Table 2. The AAC score was significantly negatively correlated with apple intake, but not with total fruit intake or other specific fruits. Total fruit and apple intake were then divided into tertiles and the difference in AAC score across the groups was explored using ANCOVA. In multivariable adjusted models AAC score was 4.16 ± 0.27, 3.98 ± 0.28 and 3.54 ± 0.28, for apple intake tertiles 1 (0–15 g/day), 2 (16–54 g/day) and 3 (>55 g/day), respectively (*p* = 0.04). Additional adjustment for total flavonoids, fibre, potassium, magnesium, vitamin C, total vegetable and saturated fat intake only marginally changed the observed association between apple intake and AAC24 score (*p* = 0.05). The AAC score was not significantly different across tertiles of total fruit or other individual fruit intakes (*p* > 0.20).

**Figure 1:**
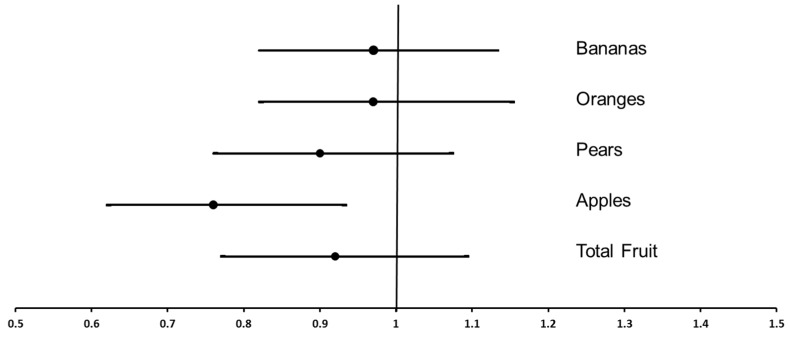
The odds ratio and 95% confidence interval of severe abdominal aortic calcification for each SD increase in total fruit, apple, pear, banana and orange intake. Multivariable adjusted for age, BMI, energy intake, energy expended in physical activity, alcohol consumption, prevalent atherosclerotic vascular disease, previous type 2 diabetes, use of antihypertensive medication, use of cholesterol-lowering medication (statins), and smoking status.

**Figure 2:**
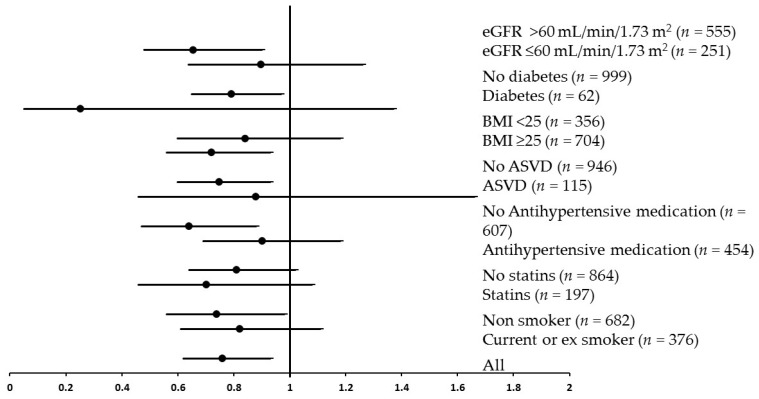
The odds ratio and 95% confidence interval of severe abdominal aortic calcification for each SD increase in apple intake (50 g/day) stratified by estimated glomerular filtration rate (eGFR) diabetes, BMI, prevalent atherosclerotic vascular disease (ASVD), use of antihypertensive medication, use of statins and smoking status. Multivariable adjusted for age, BMI, energy intake, energy expended in physical activity, alcohol consumption, prevalent atherosclerotic vascular disease, previous type 2 diabetes, use of antihypertensive medication, use of cholesterol-lowering medication (statins), and smoking status. There was no significant interaction of apple intake with any of the strata for these parameters in predicting severe AAC (*p* > 0.05).

In order to further explore the relationship of fruit intake with the risk of severe calcification in the abdominal aorta, the women classified as having severe calcification (AAC > 5; *n* = 196) were compared to the women without severe calcification (AAC = 0–5; *n* = 865; Figure 1). In age-adjusted models, each SD increase in apple intake (50 g/day; approximately half of a small apple) was associated with a 26% lower odds of having severe AAC (OR: 0.74 (0.61, 0.90), *p* = 0.003). In multivariable-adjusted models, each SD increase in apple intake was associated with a 24% lower odds of having severe AAC (OR: 0.76 (0.62, 0.93), *p* = 0.009; Figure 1). There was no attenuation of this relationship after adjustment for total flavonoids, fibre, potassium, magnesium, vitamin C, total vegetable and saturated fat intake (OR: 0.70 (0.55, 0.91), *p* = 0.008). Total fruit intake and other individual fruits were not associated with increased odds of having severe AAC in age-adjusted or multivariable adjusted models (Figure 1). The OR for severe AAC in relation to apple intake was then stratified according to BMI, health status and the use of medications (Figure 2). There was no significant interaction of apple intake with any of the strata for these parameters in predicting severe AAC (*p* > 0.05).

## 4. Discussion

The population included in this study were women over the age of 70. Although pre-menopausal women experience a lower rate of cardiovascular disease in comparison to men, after menopause the risk of CVD increases significantly [40]. For this reason it is important to investigate modifiable risk factors for CVD in this population, and their relationship with sub-clinical atherosclerosis, which is a major underlying contributor to CVD events. Higher fruit intake is consistently associated with lower risk for all-cause and disease-specific mortality in observational cohort studies [5]. It is also associated with a reduced risk of ischemic heart disease and stroke incidence [4].

We found that calcification of the abdominal aorta was negatively associated with apple intake, but not with the intake of pears, oranges and other citrus or bananas. A 1 SD (50 g/day) increase in apple intake was associated with 24% lower odds of having severe AAC. An apple usually weighs between 100 and 150 grams. These relationships were sufficiently robust to remain significant in multivariable-adjusted models that included factors known to be associated with a healthier diet, as well as nutrients in apples that may have potentially mediated the effects.

Abdominal aortic calcification is remarkably common, particularly in the elderly and those with chronic kidney disease [41,42]. Unlike coronary calcification, it can be made up of medial calcification, intimal calcification or a mix of both [43]. Using a simple scoring system for AAC, a relationship has been shown between the presence of calcific deposits in the abdominal aorta and increased CVD risk [44,45]. In our cohort, AAC was positively associated with age and alcohol intake and was higher in those with a history of atherosclerotic vascular disease, those taking antihypertensive medication or cholesterol lowering medication and in current and previous smokers. This was expected as elevated cholesterol, hypertension, aging and smoking are amongst the most widely accepted risk factors for atherosclerosis [46,47,48]. In addition, an increase in coronary artery calcification (CAC) has been observed with high dose and long-term statin therapy, but this CAC progression is likely to represent plaque repair rather than continuing plaque development [49].

Apple consumption has been related to benefits on lipid metabolism [50,51], blood pressure and vascular function [52], inflammation [50,53], oxidative stress [54,55] and type 2 diabetes [16,56]. These effects may be moderated by the microflora in the intestines [57]. It has been suggested that the beneficial effects of a diet rich in fruits can be attributed, in part, to flavonoids [16]. Apples, being one of the most ubiquitous fruits consumed worldwide, are often an important contributor to total flavonoid intake. In an acute, human intervention study, a flavonoid-rich apple blend improved measurements of endothelium-dependent vasodilation and decreased systolic blood pressure compared to a low flavonoid apple control [52], supporting the critical role of flavonoids in mediating the protective effects of apples. The main flavonoids found in apples are quercetin, phloridzin, chlorogenic acid and epicatechin [58]. Quercetin has been shown to reduce blood pressure in hypertensives [59] and there is evidence for its beneficial effects on atherosclerosis [60,61]. In the Finnish Mobile Clinic Health Examination Survey, it was found that high quercetin intake was associated with lower mortality from ischaemic heart disease, with much of the quercetin coming from apples [16]. In the current cohort of elderly women we have shown that quercetin intake was associated with a 40%–50% lower risk of atherosclerotic vascular disease mortality [62]. Indirect evidence also supports beneficial effects of other flavonoids found in apples in impeding the spread of inflammation [63]. Phloridzin prevents stimulated expression of endothelial adhesion molecules and reduces platelet aggregation [64], quercetin [59] and chlorogenic acid [65] prevent oxidation of LDL (low-density lipoprotein), and epicatechin can attenuate the development of atherosclerosis [66].

Other components of apple might also attenuate the development of atherosclerosis. In a recent study by Zhao *et al.*, following the consumption of one apple/day for four weeks, plasma concentrations of oxidized LDL/beta2-glycoprotein I complex (oxLDL-β2GPI), a proposed contributor to atherosclerosis, were decreased considerably [67]. A smaller benefit was seen with capsules of apple polyphenol extract [67], indicating that the beneficial effects of apples are due to factors other than polyphenols. In a study by Ravn-Haren *et al.*, decreases in serum total- and LDL-cholesterol were only observed after intervention with whole apple, apple pomace or cloudy apple juice compared to clear apple juice [68]. They conclude that the beneficial effects of apples as a whole could come from a synergistic effect between apple polyphenols and fibre, an interaction which may be mediated by the gut microflora [57]. Indeed higher fibre intake, particularly fruit fibre, has been associated with lower risk of cardiovascular disease [69,70,71]. Pectin, the most abundant fibre in apples, has been shown to reduce circulating cholesterol concentrations [72]. Additionally, there is evidence that magnesium [73], potassium [74] and vitamin C [75], all contribute to the beneficial effects of fruit against CVD. In the current study adjustment for these nutrients alongside fibre and flavonoids had little impact on multivariate adjusted effects indicating that a combination of components may be involved.

### Limitations

The observational cross-sectional nature of this study is a limitation as it only allows for associations, rather than cause and effect, to be tested. In this study abdominal aortic calcification was assessed using a bone densitometer which is comparable to standard radiographs but is less sensitive than CT based assessment of AAC. Despite lower sensitivity, there is good agreement between the methods [76]. We were unable to distinguish between intimal and medial aortic calcification, with the latter occurring independently of atherosclerosis [43]. Although baseline cardiovascular and dietary confounders were adjusted for in the statistical models, residual confounding cannot be overlooked. Additionally, it is important to note that lifestyle behaviours tend to cluster; a higher intake of fruit is also associated with a better overall diet, not smoking, increased levels of physical activity, and being of a higher socioeconomic status. Furthermore, the use of a food frequency questionnaire can lead to an inaccurate representation of flavonoid intake due to recall bias and the fact that not all flavonoid sources are included in the questionnaire. Although the questionnaire approach has been vastly improved by the use of new databases of flavonoid composition, there is a large variation in flavonoid content between foods of the same type due to factors such as variety, storage and cooking. However, this method remains the best available for assessment of long-term habitual flavonoid intakes. The findings of this study are also limited to elderly women and therefore caution should be taken in generalizing the results to other populations.

## 5. Conclusions

In an analysis of a cohort of 1052 women over the age of 70 years, apple intake was dose-dependently associated with the severity of AAC. Furthermore, after multivariable adjustments for health and lifestyle factors, we found that for each 50-gram increase in apple intake per day, the odds of severe AAC decreased by 24%. It appears as though there is some truth behind the 19th century health promotion message “an apple a day keeps the doctor away”.

## Figures and Tables

**Table 1:** Baseline characteristics.

Characteristic	Mean ± SD ^1^
Age (year)	75.1 ± 2.7
BMI (kg/m^2^)	27.2 ± 4.7
Energy intake (kJ/day)	7244 ± 2172
Physical activity (kJ/day)	595 ± 658
Alcohol (g/day)	6.8 ± 9.9
Prevalent ASVD (*n* (%))	172 (11.8)
Previous type 2 diabetes (*n* (%))	87 (6)
BP lowering medication use (*n* (%))	630 (43.3)
Statin use (*n* (%))	270 (18.5)
Smoked ever (*n* (%))	538 (37.2)
AAC score ( x̃ (IQ range))	2 (4)
Fruit intake	
Total ( x̃ (IQ range) g/day)	230.9 (205.5)
Apple ( x̃ (IQ range) g/day)	31.8 (60.3)
Pear ( x̃ (IQ range) g/day)	11.3 (33.1)
Orange ( x̃ (IQ range) g/day)	35.8 (80.8)
Banana ( x̃ (IQ range) g/day)	44.6 (53.7)

ASVD, atherosclerotic vascular disease; AAC, abdominal aortic calcification; BP, blood pressure; IQ, interquartile. ^1^ Values presented as mean ± SD unless otherwise stated

**Table 2:** Spearman’s Correlation of Abdominal Aortic Calcification (AAC) scores at baseline with total fruit and individual fruit (apples, pears, oranges and bananas) consumption at baseline (g/day).

AAC Score	Correlations				
	Total fruit	Apples	Pears	Oranges	Bananas
Spearman’s rho (*ρ*)	−0.061	−0.089	−0.016	−0.012	−0.035
*p*-Value	0.05	<0.01	0.60	0.70	0.25
